# Assessing a Novel Method to Reduce Anesthesia Machine Contamination: A Prospective, Observational Trial

**DOI:** 10.1155/2018/1905360

**Published:** 2018-02-04

**Authors:** Chuck J. Biddle, Beverly George-Gay, Praveen Prasanna, Emily M. Hill, Thomas C. Davis, Brad Verhulst

**Affiliations:** ^1^Department of Nurse Anesthesia, Virginia Commonwealth University, Richmond, VA, USA; ^2^Department of Anesthesiology, Virginia Commonwealth University Medical Center, Richmond, VA, USA; ^3^Department of Clinical Lab Sciences, Virginia Commonwealth University, Richmond, VA, USA; ^4^Department of Psychology, Michigan State University, East Lansing, MI, USA

## Abstract

**Background:**

Anesthesia machines are known reservoirs of bacterial species, potentially contributing to healthcare associated infections (HAIs). An inexpensive, disposable, nonpermeable, transparent anesthesia machine wrap (AMW) may reduce microbial contamination of the anesthesia machine. This study quantified the density and diversity of bacterial species found on anesthesia machines after terminal cleaning and between cases during actual anesthesia care to assess the impact of the AMW. We hypothesized reduced bioburden with the use of the AMW.

**Methods:**

In a prospective, experimental research design, the AMW was used in 11 surgical cases (intervention group) and not used in 11 control surgical cases. Cases were consecutively assigned to general surgical operating rooms. Seven frequently touched and difficult to disinfect “hot spots” were cultured on each machine preceding and following each case. The density and diversity of cultured colony forming units (CFUs) between the covered and uncovered machines were compared using Wilcoxon signed-rank test and Student's *t*-tests.

**Results:**

There was a statistically significant reduction in CFU density and diversity when the AMW was employed.

**Conclusion:**

The protective effect of the AMW during regular anesthetic care provides a reliable and low-cost method to minimize the transmission of pathogens across patients and potentially reduces HAIs.

## 1. Introduction

Healthcare associated infections (HAIs) are a major source of preventable illness, with approximately 2 million occurring in US hospitals every year [1] at a cost approaching 4.5 billion dollars [2]. This poses a serious safety concern associated with a significant increase in patient morbidity, mortality, and healthcare costs. Anesthesia machines are known to be active reservoirs for pathogens contributing to the burden of HAIs [1–4]. The transmission of bacterial pathogens via anesthesia machines occurs both during and between patient cases due to high task density, frequent contact with body fluids, invasive procedures, and provider errors, such as omission of adequate hand hygiene. All of these risk factors are performed within the small confines of the anesthesia work area. These issues have been identified as one of the causes of 30-day postoperative surgical site infection, blood stream infection, central line infection, and ventilator acquired pneumonia in patients undergoing surgery [1–4]. The source of this contamination could be the transfer of organisms from the patient themselves or from workstation equipment reservoirs such as the anesthesia machine [2, 4].

The design of the anesthesia machine makes routine disinfection, sterilization, and cleaning difficult, with complete decontamination all but impossible in daily practice. The adjustable pressure limiting (APL) valves, gas flowmeters, and the agent vaporizer dials of the anesthesia machine are common reservoirs for enterococci and other pathogenic bacterial species [2–11]. Institutional cleaning protocols vary widely and are ineffective in eliminating pathogenic contamination, leading some infection control epidemiologists to refer to the “fecal patina in the anesthesia work area” [6]. In the range of operative settings nationwide, there is likely great variability in cleaning practices. Pathogenic microorganisms are known to survive on the anesthesia machine after standardized, routine cleaning, with bacterial burden reduced but not eliminated, even after accelerated cleaning practices are initiated [5–8]. In simulations, we and others have demonstrated that routine, between-case cleaning is inadequate in removing a fluorescent marker serving as a surrogate pathogen, the inoculum persisting as a potential infection source for a subsequently cared-for patient [6–9]. Current best cleaning practices fail in achieving full decontamination and thus may place subsequent patients at considerable risk of cross contamination.

To reduce the prevalence of pathogens in the operating room, it may be possible to utilize a disposable, engineered barrier that prevents the contamination of the anesthesia machine during surgery. Use of an ultrathin polyvinyl chloride (PVC) transparent anesthesia machine wrap (AMW) is akin to wearing gloves during procedures and disposing of them after each use, though ergonomics and clinical efficacy have not been systematically studied. An AMW is relatively inexpensive, costing approximately $35.00, with price factors depending on the specific anesthesia machine in a given OR (http://www.anesthesiahygiene.com, Miami, FL). Its use may reduce horizontal transfer of pathogens to the machine, decrease the potential for machine-vectored contamination in ensuing patients, and improve the efficacy of current cleaning methods. The use of an AMW is intended to supplement universal precautions (UP) in the healthcare setting. Healthcare workers are mandated to practice UP to prevent horizontal and vertical transmission of pathogens. The Occupational Safety and Health Administration (OSHA) describes, as part of a UP program, that, in addition to self-protection, practitioners “use engineering and work practice controls to limit exposure” [12].

The primary aim of this research was to examine the effectiveness of the AMW in a clinical setting by testing the following hypotheses: (1) in operating rooms where the AMW is used there will be fewer colony forming units (CFUs) on the anesthesia machine compared to machines without the AMW and (2) in rooms where the AMW was used there will be less diversity in microbial species compared to machines without the AMW.

## 2. Materials and Methods

### 2.1. Description of the Anesthesia Machine Wrap (AMW)

**Figure 1:**
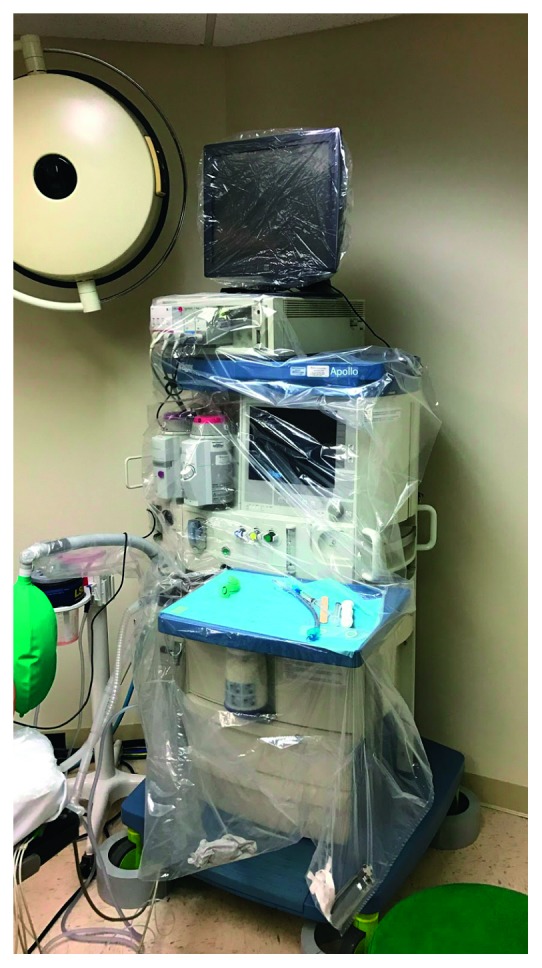
AMW displayed on a functioning machine.

The AMW is shown in Figure 1 (http://www.anesthesiahygiene.com, Miami, FL) and was used on Fabius, GS machines (Drager, Telford, PA) for all trials. In an unpublished pilot simulation study, we determined that the use of the AMW did not impede clinical performance and was readily accepted by a diverse group of experienced anesthesia providers. The AMW has strategically located adhesive strips that allow the wrap to securely adhere to the anesthesia machine. It can be fitted onto the machine in less than two minutes and can be removed in seconds avoiding personal contamination, much like a surgical drape. It also has three pouches that may be used for equipment, drug vials, or trash.

### 2.2. Study Procedure

Following institutional review board approval at Virginia Commonwealth University Medical Center, the AMW was applied to the anesthesia machines in conveniently selected operating rooms where general anesthesia was provided to adult patients undergoing open abdominal surgery; there was no change or influence on surgical or anesthetic management in any case. The AMW was used in 11 surgical cases (intervention group) and absent in 11 other surgical cases (control group) that were consecutively assigned to two general surgical operating rooms on the three days that the trial was conducted. This sample size was determined to be sufficient to reliably identify statistically significant differences in both density and diversity of CFUs between the two groups. Based on prior simulated pilot study results, a large effect size (approximately 1.25 SD units) was assumed, with one-tailed hypothesis tests. A sample size of 10 observations per condition would provide >80% power to detect a significant difference in contamination; a sample size of 11 per condition was obtained.

Anesthesia providers in the control and intervention (AMW) arms of the study were unaware of the study purpose. Those in the intervention (AMW) were only told that their machines were outfitted with a wrap and their perceptions of its ease of use would be queried at case end. Microbial cultures were obtained when both control room and intervention room providers were not present.

Based on the findings of previous research, microbial analysis was performed on samples collected from seven cultured “hot spots” for bacterial contamination [7, 13, 14]. We operationalized “hot spots” as targeted areas on the anesthesia machine that were likely to be contaminated by providers' clinical care activities and were difficult to clean/disinfect using routine procedures. The seven “hot spots” were as follows:

- Vaporizer dial
- Patient monitor control panel
- Mechanical ventilator control knob
- Oxygen flowmeter control knob
- Mouse control for electronic medical record
- Keyboard for the electronic medical record
- APL valve control knob.

Culture samples for each “hot spot” were obtained prior to first surgical case of the day in the operating rooms, after the anesthesia provider completed the machine check. This provided the baseline for bacterial contamination in each room (control and AMW rooms). Following collection of samples for culture, the AMW was applied on the anesthesia machine in the study arm; no further manipulation or intervention was performed in the “control” room (no AMW application). After the completion of the surgery and once the patient was transported out of the operating room, the AMW was removed from the anesthesia machine, and samples for culture were obtained from the seven sites listed above. This was performed in all of the operating rooms prior to the routine, between-case cleaning of the anesthesia machines in order to measure any intraoperative contamination that occurred as a consequence of caring for the current surgical patient.

Once the anesthesia machine was cleaned in the standard manner by the anesthesia technicians (who were unaware of the study purpose), the machine in the intervention room had a new AMW applied in anticipation of the arrival of the second surgical patient. No alteration from routine practice was conducted on the anesthesia machine in the control arm of the study (no AMW).

### 2.3. Routine Anesthesia Machine Cleaning Protocol

Cleaning protocols for anesthesia equipment are highly variable from institution to institution, with no formalized set of national guidelines. This study assessed the AMW at an institution where cleaning and disinfecting processes follow a strict protocol by highly trained anesthesia technicians cleaning/disinfecting the anesthesia equipment based on both institutional and anesthesia machine manufacturer standards. OxyCide™ (EcoLabs, St. Paul, MN) disinfecting chemicals and wipes are used on each machine at case end and at terminal cleaning at the end of the day. Additionally, an ultra violet (UV) light robot is used following terminal cleaning in each operating room at least once a week.

### 2.4. Performing Cultures

The seven target surfaces (hot spots) on the anesthesia machine in the intervention and control rooms were cultured for microbiological analysis using ESwabs™ (COPAN Diagnostics Inc., Waltham, MA) by a clinical microbiologist. The tip of a sterile flocked nylon swab was immersed in tween (a nonionic surfactant) then pressed against the wall of the tube to remove excess solution. The target surface was swabbed using a rotating and twisting motion. Each respective swab was immediately placed in a transport system containing one milliliter of liquid Aimes media. Upon arrival to the laboratory, each ESwab was vortexed for approximately two minutes, and 100 microliters of each respective sample was inoculated to sheep blood agar and MacConkey agar (Copan Diagnostics, Inc., Corona, CA). The inoculum was evenly distributed using a cell spreader, and agar plates were incubated at 35°C for 48 hours. Following incubation, the sheep blood agar and MacConkey agar plates were observed for growth. Each colonial morphotype present on the media was identified by gross examination, and CFUs were recorded. Organisms were subcultured to SBA for isolation and identification. For the purpose of this study, organisms were identified based on colonial morphology, Gram stain, and rapid spot tests such as catalase, Staphaurex™ Plus latex agglutination, pyrrolidonyl arylamidase, Remel™ Microdase discs, indole, and oxidase (ThermoFisher Scientifics, Inc., Middletown, VA).

### 2.5. Statistical Analysis

Wilcoxon signed-rank tests were used to test the effectiveness of the AMW for reducing the density of microbiological contamination between the cultured CFUs in covered and uncovered conditions. The Wilcoxon signed-rank test is a nonparametric analog of the *t*-test that accounts for major deviations from normality. To examine differences in the diversity of the microbacterial contaminants, a standard *t*-test was used. Differences in the density and diversity of CFUs were tested at both the global level and for each specific hot spot. To account for multiple testing, in addition to the nominal *p* values for each test, we present Bonferroni-adjusted *p* values and false discovery rate (FDR) *q* values. Bonferroni-adjusted *p* values are known to be extremely conservative, thus greatly increasing the likelihood of a type II error. FDR *q* values are similar to adjusted *p* values, but quantifying the likelihood of observing a false positive result from an observation equally or more extreme than that in question. Therefore, a *q* value of 0.022 would imply that 2.2% of the observations at least this extreme is expected to be false positives.

## 3. Results

**Figure 2:**
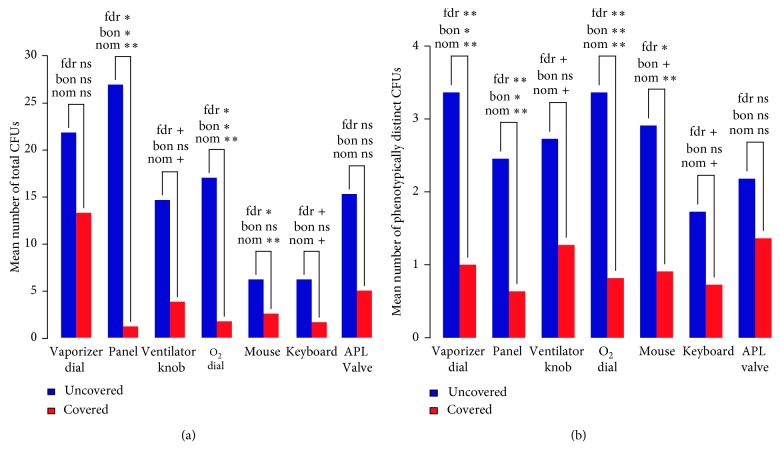
Graphic depiction of CFUs and new species added to each of the hot spots. (a) Density of CFUs. (b) Diversity of CFUs. (a) depicts the differences in the density of CFUs between the AMW and no AMW conditions for each “hot spot”. At each “hot spot,” there were fewer overall CFUs in the AMW cases compared to those where the AMW was not employed. Likewise, there are fewer additional bacterial species added to the individual hot spots, suggesting that the AMW was protective against contamination of the anesthesia machine from previous patients cared for, likely by preventing the anesthesia provider from serving as a vector. To account for multiple testing, nominal unadjusted *p* values, Bonferroni-adjusted *p* values, and FDR *q* values are presented. CFUs = colony forming units; AMW = anesthesia machine wrap; fdr = false discovery rate *q* value; bon = Bonferroni-adjusted *p* value; nom = nominal *p* value; +=*p* < 0.10; ∗ = *p* < 0.05; ∗∗ = *p* < 0.01; ns = not significant (*p* > 0.10).

The anesthesia machines covered with the AMW had a statistically significant reduction in global density of CFUs recorded across all hot spots with fewer CFUs compared to the uncovered machines (means of the total CFUs on the Control and AMW Machines were 108.0 and 29.2, resp.; *p*_nominal_ = 0.008, *p*_bonferroni_ = 0.066, *q*_fdr_ = 0.022). As can be seen in Figure 2(a), there was also a consistent reduction in the number of CFUs across the specific hot spots with the AMW in situ, with statistically significant differences for the monitor control panel, the oxygen flowmeter knob, and the record keeper mouse. These data indicate that the use of the AMW was strongly associated with a reduced prevalence of microorganisms.

There was a statistically significant decrease in the diversity of CFUs across all hot spots with the covered anesthesia machines, with the exception of the APL valve machines (means of the total number of distinct CFUs on the Control and AMW Machines were 18.7 and 6.7, resp.; *p*_nominal_ = 0.0004, *p*_bonferroni_ = 0.003, *q*_fdr_ = 0.003).  Figure 2(b) presents the results for the diversity of CFUs at the individual hot spots. As can be seen, there was a consistent reduction in the number of phenotypically unique CFUs across sites with the AMW in situ, with statistically significant differences for the vaporizer dial, the monitor control panel, the oxygen flowmeter knob, and the record keeper mouse. These data indicate that the use of the AMW was strongly associated with a reduced diversity of microorganisms.

**Figure 3:**
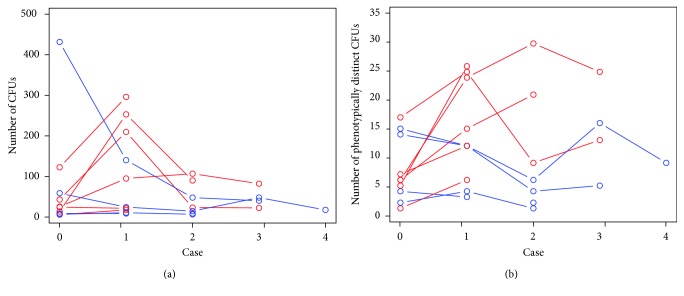
The global density and diversity of CFUs across time. (a) Density of CFUs. (b) Diversity of CFUs. (a) presents a schematic depiction of the density (quantity), and (b) presents a depiction of the diversity (phenotypically distinct) of CFUs across time. Each line represents cases in a particular OR. The blue lines depict cases in which the anesthesia machine was covered with the AMW, and the red lines depict cases where an AMW was not employed. In cases where the AMW was used, there was a decrease or plateauing of the density of CFUs, while in those cases without the AMW there was general increase in the density of CFUs. Relatedly, in cases without the AMW, additional, phenotypically distinct species were added to the anesthesia machine, suggesting ongoing contamination with the anesthesia provider as vector. Note that in cases where the AMW was used, there tended to be a reduction or stabilization in bacterial species, with one exception, which suggests contamination possibly during the cleaning or machine preparation. CFUs = colony forming units; AMW = anesthesia machine wrap.

Because several of the ORs were used repeatedly, it is possible to track the trajectory of the density and diversity of the bacterial contaminates over time.  Figure 3 graphically displays the microbial characteristics over the course of surgical case progression revealing a decrease or a stabilization of species in the AMW cases and an increase in species and density over the course of a day in the control room. We interpret these data to indicate that the use of the AMW prevented the introduction of new bacterial species to the anesthesia machine as a result of the anesthesia care rendered from one patient to the next.

Figure 3(a) demonstrates a decrease in CFUs from one case to another in the machines that had the AMW in place (blue lines). Likely this indicates that the initial burden of CFUs decreased with subsequent cleaning of the machine after each case, and that no additional bioburden was added due to the presence of the AMW. Alternatively, the unwrapped machines generally experienced increased CFUs likely due to the continued proliferation of existing species or the addition of new species resulting from contamination from a patient being cared for.

Figure 3(b) reveals an increase in new species that were added to the unwrapped anesthesia machines, suggesting that patients being cared for, with the anesthesia provider as vector, added to the diversity of bioburden despite cleaning of the machine at case end (red lines). The blue lines reveal a general decrease in species diversity when the AMW was employed, suggesting that the machines were protected from inoculation during patient care. One room in the AMW group (blue line) experienced an increase in new species that might have occurred during between-case cleaning of the machine or from the provider readying the machine for the upcoming case.

## 4. Discussion

This is the first intraoperative evaluation of an AMW using bacteriological assessment during actual clinical care. The study results show that (1) despite state of the art cleaning protocols, the anesthesia machine remains a reservoir of bacterial species; (2) intraoperative use of an AMW results in statistically significant reduction in CFUs on the anesthesia machine compared to care delivered without an AMW; and (3) intraoperative use of an AMW results in a statistically significant reduction in the introduction of new species of bacteria onto the anesthesia machine compared to care delivered without an AMW. These results lead to the conclusion that the AMW reduced the density and diversity of microorganisms in the anesthesia workstation.

**Figure 4:**
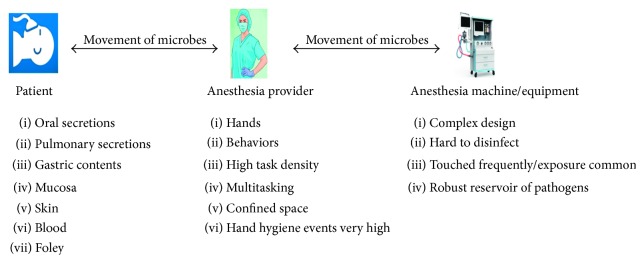
Horizontal transfer of microbes in the anesthesia work area.

The importance of engaging in UP during anesthesia care cannot be overemphasized due to the certainty of personnel and equipment coming in contact with biologic material and routinely observed suboptimal levels of hand hygiene compliance, with the anesthesia machine acting as an epicenter for intraoperative pathogens [2–5, 11, 15–17]. As depicted in Figure 4, anesthesia care providers are in nearly constant, repetitive contact with the patient (e.g., skin, mucosa, and oral secretions), the anesthesia workstation (e.g., the vaporizer dial, APL valve, and ventilator knob), and then the patient again (e.g., placing intravenous lines, obtaining blood specimens, and intravenous drug administration). Accordingly, the anesthesia workstation is an essential intermediate source of contamination, and the AMW may reduce the proliferation of pathogens across cases.

There has been a lag in identifying the anesthesia provider as a vector in the genesis of operative infection, largely because a blood stream infection, surgical site infection, or a pulmonary infection may not manifest for many hours (or days) after an inoculation occurred. By that time, the anesthesia provider's role may not be considered. Only recently has the biologic plausibility of an anesthesia provider as a vector been identified. Emerging evidence confirms the horizontal movement of pathogens as shown in Figure 4. In fact, transmission of bacteria species of all kinds, including vancomycin-resistant enterococci (VRE), MRSA, and other pathogens occurs frequently and within just a few minutes of care delivery in the anesthesia workstation [11, 15, 16].

This single center study constitutes a small sample size even though it was sufficiently powered to detect large differences in the presence of bacteria as a function of AMW use. We recognize that our study does not reveal the origin and direction of movement of microbes. The clinical outcomes effect of the density, and diversity of contamination was not measured in the study. Baseline samples were potentially contaminated since they were collected after the machine check procedure by the anesthesia provider. Additionally, some providers may have better hygienic practices than others; we neither monitored nor controlled to best depict real world practice.

While the AMW may not have a place in routine intraoperative care, this report suggests that there are scenarios where the need for enhanced protection may be indicated, for example, when caring for a patient with a known virulent infectious process (e.g., hepatitis, prion disease, MRSA, VRE, CRE, clostridium difficile, HIV, and Ebola) or when the risk of vertical transmission to the next patient of may have catastrophic consequences (e.g., joint arthroplasty, cardiac surgery, and the immunocompromised patient). While UP are an absolute standard of care, lapses in hand hygiene, difficulty in decontamination of complex equipment, and the ergonomics of the anesthesia workstation urge that we develop better methods to reduce infectious iatrogenesis.

Achieving thorough hand hygiene and equipment decontamination may be difficult, if not nearly impossible, despite the high contamination potential in the intraoperative period. A systematic approach, in line with OSHA recommending engineering environmental and practice controls, utilizing a disposable equipment barrier seems logical. We studied the efficacy of the AMW at a site where UP, equipment disinfection, and appreciation for the risk of nosocomial infection at the hands of the anesthesia provider are likely optimal in the real world of clinical practice. We suspect that at sites where there is greater variability in these domains there will be greater degrees of microbial contamination.

The results from this study do not suggest that the AMW is a panacea for contamination, but rather that it could be used in conjunction with UP to minimize the risk of infection-based complications after surgery and should not provide users with a false sense of cleanliness. In this vein, three points must be made. First, the AMW will not prevent the spread of airborne pathogens that may persist regardless of the use of any type of protective barrier. Second, standard cleaning procedures will be effective for macroscopic contaminants that are visible to the naked eyes of the cleaning crew, such as major blood spray. Third, caution is necessary when removing the AMW to avoid contaminating the operating room with what was deposited on the AMW during surgery. We expect that the AMW will be effective in reducing microscopic contaminants that cannot be seen by the naked eye and/or are deposited on textured surfaces that are difficult to clean.

A list of the bacteria that was cultured in for each hot spot is presented in Table 1. It is important to note that while the bacterial cultures we found in the current study were relatively benign, this does not imply that all organisms that may exist in the OR will always be benign. The prevalence of these organisms is relatively rare, and we would not expect to see them in a study of this size. Across a large number of surgeries, however, even relatively rare pathogens will occasionally be observed. The AMW is intended to minimize the transmission of these relevant, malignant pathogens that may cause serious HAIs.

## 5. Conclusion

Considering that use of the AMW entails negligible risk, is low cost, and is efficacious in preventing contamination of the anesthesia machine, this study identifies an opportunity to improve patient safety. The benefits of such a device may be significant and should be contemplated, particularly for selected cases where risk of microbial exposure is deemed greatest. Future designs of anesthetic machines may need to facilitate easier use of barrier precautions whilst maintaining functionality.

## Figures and Tables

**Table 1:** List of bacterial species cultured on the anesthesia machine.

Hot spot	Organisms recovered
Vaporizer dial	CoNS, *Bacillus* species, *Stomatococcus* species, *Corynebacterium* species, *Streptococcus* species, *Staphylococcus aureus*
Oxygen flowmeter knob	CoNS, *Bacillus* species, *Micrococcus* species, *Corynebacterium* species, *Streptococcus* species
APL valve control knob	CoNS, *Bacillus* species, *Micrococcus* species, *Corynebacterium* species
Record keeper mouse	CoNS, *Bacillus* species, *Stomatococcus* species, *Streptococcus* species
Monitor control panel	CoNS, *Bacillus* species, *Micrococcus* species, *Corynebacterium* species
Ventilator control knob	CoNS, *Bacillus* species, *Corynebacterium* species, *Streptococcus* species
Keyboard	CoNS, *Bacillus* species, *Corynebacterium* species

APL, adjustable pressure limiting; CoNS, coagulase-negative staphylococci.
